# Accuracy of diagnostic imaging modalities for peripheral post-traumatic osteomyelitis – a systematic review of the recent literature

**DOI:** 10.1007/s00259-017-3683-7

**Published:** 2017-04-27

**Authors:** Geertje A. Govaert, Frank F. IJpma, Martin McNally, Eugene McNally, Inge H. Reininga, Andor W. Glaudemans

**Affiliations:** 1 Department of Surgery, Subdivision of Trauma Surgery, University of Groningen, University Medical Center Groningen, Groningen, The Netherlands; 2 Department of Trauma Surgery, University of Utrecht, University Medical Center Utrecht, Room number G.04.228, P.O. Box 85500, 3508 GA Utrecht, The Netherlands; 3 The Bone Infection Unit, Nuffield Orthopaedic Centre, Oxford University Hospitals NHS Foundation Trust, Oxford, UK; 4 Oxford Musculoskeletal Radiology, Oxford, UK; 5 Department of Nuclear Medicine and Molecular Imaging, University of Groningen, University Medical Center Groningen, Groningen, The Netherlands

**Keywords:** Post-traumatic osteomyelitis, Fracture related infection, Fracture, Osteosynthetic material, Ostheosynthesis, Open reduction and internal fixation (ORIF), Diagnostic imaging, CT scan, MRI, FDG-PET, White blood cell scintigraphy, Antigranulocyte antibody scintigraphy

## Abstract

**Aims:**

Post-traumatic osteomyelitis (PTO) is difficult to diagnose and there is no consensus on the best imaging strategy. The aim of this study is to present a systematic review of the recent literature on diagnostic imaging of PTO.

**Methods:**

A literature search of the EMBASE and PubMed databases of the last 16 years (2000–2016) was performed. Studies that evaluated the accuracy of magnetic resonance imaging (MRI), three-phase bone scintigraphy (TPBS), white blood cell (WBC) or antigranulocyte antibody (AGA) scintigraphy, fluorodeoxyglucose positron emission tomography (FDG-PET) and plain computed tomography (CT) in diagnosing PTO were considered for inclusion. The review was conducted using the PRISMA statement and QUADAS-2 criteria.

**Results:**

The literature search identified 3358 original records, of which 10 articles could be included in this review. Four of these studies had a comparative design which made it possible to report the results of, in total, 17 patient series. WBC (or AGA) scintigraphy and FDG-PET exhibit good accuracy for diagnosing PTO (sensitivity ranged from 50–100%, specificity ranged from 40–97% versus 83–100% and 51%–100%, respectively). The accuracy of both modalities improved when a hybrid imaging technique (SPECT/CT & FDG-PET/CT) was performed. For FDG-PET/CT, sensitivity ranged between 86 and 94% and specificity between 76 and 100%. For WBC scintigraphy + SPECT/CT, this is 100% and 89–97%, respectively.

**Conclusions:**

Based on the best available evidence of the last 16 years, both WBC (or AGA) scintigraphy combined with SPECT/CT or FDG-PET combined with CT have the best diagnostic accuracy for diagnosing peripheral PTO.

## Introduction

Post-traumatic osteomyelitis (PTO), also known as ‘fracture-related’ osteomyelitis, is a feared complication for its difficult recognition, significant treatment duration and high recurrence rate. Infection can present acutely in the first few weeks after internal fixation, in a delayed manner with low-grade infection or late with infected non-union or persistent infection after fracture healing [1–3]. The incidence of deep infection after surgical fracture care is relatively high (between 1 and 19%) [4–6], depending on trauma-related risk factors such as contaminated open fractures, damage control procedures and concomitant soft tissue injuries. Early treatment of an acute infection can prevent progression to established PTO but this condition still affects 2–4% of all patients undergoing an open reduction and internal fixation of an open or closed fracture [7].

The key for a successful treatment of PTO is a prompt and accurate diagnosis. However, this diagnostic process in particular is challenging [7–19]. Many imaging modalities such as magnetic imaging resonance (MRI), three-phase bone scintigraphy (TPBS), white blood cell (WBC) scintigraphy, antigranulocyte antibody (AGA) scintigraphy, fluorodeoxyglucose positron emission tomography (FDG-PET) and plain computed tomography (CT) are frequently used for diagnosing or excluding this condition. In the past 10 years, there has been a huge development in new camera systems, combining nuclear medicine techniques such as single-photon emission computed tomography (SPECT) and PET with radiological techniques such as CT and MRI. Although these hybrid camera systems (SPECT-CT, PET-CT or PET-MRI) may lead to better localisation of the infection and, as a consequence, to better diagnostic accuracy rates, their diagnostic value for PTO has not yet been established [19–21].

The aim of this study is to present a systematic review of the recent literature (from 2000 to 2016) on imaging techniques to diagnose PTO.

## Materials and method

The PRISMA (Preferred Reporting Items for Systematic Reviews and Meta-Analyses) statement [22] and its “Explanations and Elaboration” [23] were the guidance for this systematic review.

### Search strategy

Following the recommendations of the Cochrane collaborations, a computerised literature search in the PubMed and Embase databases was conducted. Included were articles in any language published between January 1^st^ 2000 and December 31^st^ 2016. Search terms (Table 1) were defined by two authors with the assistance of a professional information retrieval specialist. The Cochrane Library [24] was checked for reviews on diagnostic imaging modalities for osteomyelitis. In addition, references of included studies and of relevant review articles, editorials and/or commentaries of the last 16 years were scrutinized for additional articles to be included.

**Table 1:** Search strings for Pubmed and Embase

PUBMED
("Osteomyelitis"[Mesh] OR "Osteitis"[Mesh] OR ("Surgical Wound Infection"[Mesh] AND bone*[tiab]) OR osteomyelitis[tiab] OR osteitis[tiab]) AND ("Diagnostic Imaging"[Mesh] OR "Magnetic Resonance Imaging"[Mesh] OR "Tomography, X-Ray"[Mesh] OR "Tomography, Emission-Computed"[Mesh] OR "Radionuclide Imaging"[Mesh] OR "Positron-Emission Tomography"[Mesh] OR "Fluorodeoxyglucose F18"[Mesh] OR "Leukocytes/radionuclide imaging"[Mesh] OR "Technetium Tc 99 m Exametazime"[Mesh] OR diagnostic imaging[tiab] OR MRI[tiab] OR "bone scan"[tiab] OR "CT scan"[tiab] OR "computed tomography"[tiab] OR SPECT-CT[tiab] OR SPECT/CT[tiab] OR PET[tiab] OR PET/CT[tiab] OR PET-CT[tiab] OR FDG[tiab] OR fluorodeoxyglucose[tiab] OR scintigraphy[tiab]) NOT Case Reports[ptyp] AND PY: from 2000, added to Pubmed until dec2015
EMBASE
‘osteomyelitis’/mj OR ‘osteitis’/mj OR (‘surgical infection’/exp/mj AND bone*:ab,ti) OR osteomyelitis:ab,ti OR osteitis:ab,ti AND (‘diagnostic imaging’/exp OR ‘nuclear magnetic resonance imaging’/exp OR ‘tomography’/de OR ‘computer assisted tomography’/exp OR ‘emission tomography’/exp OR ‘whole body tomography’/exp OR ‘scintiscanning’/exp OR ‘fluorodeoxyglucose f 18’/exp OR (‘leukocyte’/exp/mj AND imaging) OR (‘technetium 99 m’/exp/mj AND imaging) OR ‘diagnostic imaging’:ab,ti OR mri:ab,ti OR ‘bone scan’:ab,ti OR ‘ct scan’:ab,ti OR ‘computed tomography’:ab,ti OR ‘spect-ct’:ab,ti OR ‘spect/ct’:ab,ti OR pet:ab,ti OR ‘pet/ct’:ab,ti OR ‘pet-ct’:ab,ti OR fdg:ab,ti OR fluorodeoxyglucose:ab,ti OR scintigraphy:ab,ti) NOT ‘case report’/exp AND [2000-2016]/py AND [1-1-1900]/sd NOT [31-12-2015]/sd

### Study selection

Emphasis in this review is on patients suffering from osteomyelitis of the peripheral skeleton that emerged after trauma-related injuries. Depending on the type of injury and previous treatment strategies, these could be implant-associated infections or not. For this reason, articles reporting on diagnostic medical imaging techniques for other types of bone or non-trauma-related infections were excluded. This review does not include cases of haematogenous osteomyelitis. The inclusion and exclusion criteria (Table 2) are in line with endpoints used in earlier meta-analyses on this topic [17, 25]. Only studies investigating widely available diagnostic imaging tests for osteomyelitis—which are TPBS, WBC (or AGA) scintigraphy, FDG-PET, MRI and CT scan—were eligible for this review. This study is limited to PTO of the peripheral skeleton as the upper and lower limbs are the most commonly affected anatomical regions. Furthermore, some diagnostic nuclear imaging modalities have limitations in imaging the axial skeleton, as tracers may behave differently and WBC scintigraphies are more difficult to interpret because high uptake of WBCs in the liver, spleen and bone marrow may obscure the specific uptake [19, 26]. Therefore, osteomyelitis of the axial skeleton was not assessed in this review. No concessions were made for non-trauma-related studies. Due to our desire to include the most relevant papers, we did allow a low number (<15%) of trauma-related prosthetic joint infections (PJI) and non-peripheral PTO sites provided that this was clearly stated by the authors and the data could not be extricated otherwise. If applicable, this is mentioned explicitly in the results section of this paper. The procedure for inclusion of studies was based on the recommendations by Van Tulder et al. [27].

**Table 2:** Inclusion and exclusion criteria

Inclusion Criteria
1) The study must evaluate the accuracy of radiological and nuclear imaging modalities for diagnosing PTO.
2) The study group must be at least 10 patients of 18 years and older with (suspected) PTO. In case of a mixed population, the data for this subgroup must be available independently.
3) The studied location must be in the peripheral skeleton.
4) The study must use a valid reference test (osteomyelitis was proven histologically and/or bacteriologically, and/or there was a clinical follow-up of at least 6 months in which no signs or symptoms of chronic infection were described).
5) Studies must provide sufficient details to construct a 2 x 2 contingency table expressing the results of the index tests by the disease status.
6) The study must investigate a commonly used diagnostic imaging test for PTO. These are conventional X-ray, CT, MRI, WBC scintigraphy/AGA scintigraphy (+/- SPECT/CT), bone scintigraphy (+/- SPECT/CT) and FDG-PET (+/- CT).
Exclusion Criteria
1) Non-human studies.
2) Studies that investigate non-trauma-related osteomyelitis (such as osteomyelitis due to spondylodiscitis, diabetic feet, haematogenous dissemination and pressure ulcers).
3) Studies that investigate a not commonly used diagnostic imaging test [such as ^99m^Tc-ciprofloxacin (Infecton) scintigraphy or ^68^Ga-citrate PET].

### Methodological quality assessment

The qualitative assessment of the study design was performed according to the QUADAS-2 (Quality Assessment of Diagnostic Accuracy Studies, version 2) criteria as recommended by the Cochrane Institute . QUADAS-2 is a tool for the assessment of studies of diagnostic accuracy included in systematic reviews and consists of four domains: patient selection, index test, reference standard and flow and timing [28]. Each domain is assessed in terms of risk of bias, and the first three domains are also assessed in terms of concerns regarding the applicability of a study. Authors were contacted when information regarding the quality of the study was not provided in the articles.

### Data extraction

The following data was extracted from all relevant papers: 1) author and journal; 2) year of publication; 3) type of study; 4) number of patients with PTO; 5) type of imaging modality; 6) gold standard; 7) data regarding diagnostic accuracy of the imaging modality for PTO; and 8) study limitations.

### Statistical analysis

Data analysis was conducted in line with guidelines for systematic reviews from the Cochrane Collaboration. The discriminative ability of the imaging modalities was quantified by several measures of diagnostic accuracy: sensitivity, specificity, positive and negative predictive values (PPV and NPV), positive and negative likelihood ratios (PLR and NLR) and the diagnostic odds ratio (DOR), which were calculated based on raw data reported in the papers. NPV and PPV values range between 0 and 1, and high values can be interpreted as indicating the accuracy of the diagnostic test. The NLR is the ratio of the probability of a patient with PTO having a negative test result, and a patient without PTO having a negative test result. Similarly, the PLR is the ratio of the probability of a patient with PTO having a positive test result, and a patient without PTO having a positive test result. NLR values less than 1 indicate an increase in the probability of the absence of PTO. PLR values greater than 1 indicate an increase in the probability of PTO. The DOR of a test is the ratio of the odds of positive test results in persons with the disease relative to the odds of positive test results in the non-diseased. DOR ranges from zero to infinity, with higher values indicating better discriminatory test performance. When raw data were not available, the reported sensitivity and specificity measures were presented. Data analyses was conducted using Review Manager 5.3 (version 5.3.5, The Nordic Cochrane Centre, The Cochrane Collaboration, Copenhagen, Denmark).

### Source of funding

No external funds were received in support of this study.

## Results

### Included studies

**Fig. 1:**
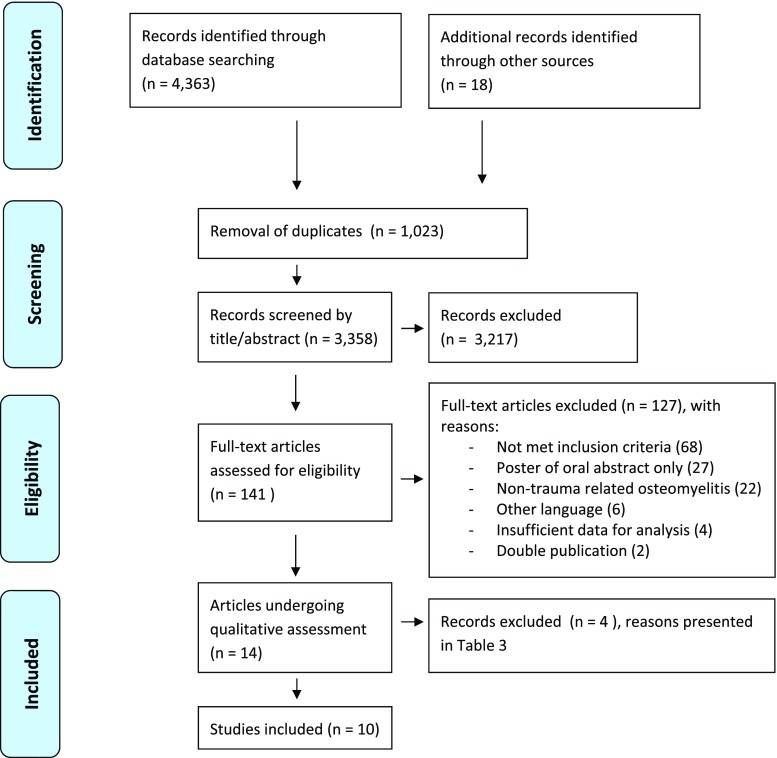
PRISMA Flow diagram

A total of 4363 articles that met the initial search criteria were identified in PubMed (n = 1846) and Embase (n = 2517). The Cochrane Library contained four entries on imaging osteomyelitis; these were all meta-analyses of which two dealt with diabetic feet [29, 30], one with chronic, mostly post-traumatic osteomyelitis [17] and one with osteomyelitis of unspecified aetiology [25]. Screening of the reference lists of these and other relevant articles found in PubMed [8, 9, 15, 18, 31–44] yielded 18 additional studies. After removal of duplicates (n = 1023), 3358 unique publications remained and were screened on title and abstract by two authors. This resulted in 141 titles, which were subsequently retrieved with the full text. The eligibility of each article was established by a group discussion until consensus was reached. One hundred and twenty-seven articles were excluded for specific reasons (Fig. 1). Eventually, 14 studies remained for further analysis [21, 45–57] and underwent qualitative assessment according to the QUADAS-2 criteria by two authors (Table 3). This resulted in four more exclusions [50, 51, 56, 57]. For this process, additional information was obtained by email from two corresponding authors [52, 55]. Finally, 10 studies [21, 45–49, 52–55] remained for inclusion in this systematic review. The inclusion process is summarized in Fig. 1.

**Table 3:**
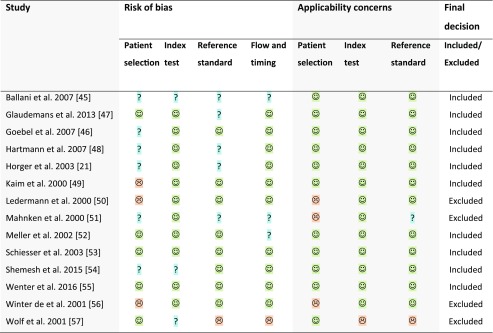
QUADAS-2 assessment of applicability

low risk;  high risk;  unclear risk

### Study quality

Table 3 presents the final results of the risk of bias assessment. The risk of bias differed between studies. In general, there were concerns regarding patient selection and reference standards. The applicability of all studies was good.

### Description of study characteristics

Four of the 10 articles [45, 46, 49, 52] had a comparative design which made it possible to include the results of, in total, 17 patient series (three studies [46, 49, 52] investigated three imaging modalities). Six studies addressed the value of FDG-PET in the diagnostic process for PTO [46, 48, 52–55], 5 studies addressed WBC or AGA scintigraphy [21, 45, 47, 49, 52], 2 studies addressed MRI [46, 49], 3 studies addressed bone scintigraphy [45, 49, 52], and one study focused on CT [46]. A schematic overview of the included studies is presented in Table 4. Due to the relatively small numbers of included studies and heterogeneity in applied diagnostic protocols, thresholds and cut-off points, pooling of data was not appropriate. Hence, results of individual studies are presented (Table 5).

**Table 4:** Schematic overview of included studies

Imaging modality	Author	Year	PTO Patients (n)	Specifics on imaging technique/ tracer	Use of hybrid imaging	Methodology	Timeframe between trauma, first symptoms of infection and diagnostic imaging
Three-phase bone scintigraphy	Ballani et al. [45]	2007	10	3-phase ^99m^Tc-MDP bone scan, 740 MBq, γ-camera with 256 x 256 matrix (pixel size ∼ 2 mm).	No	Retrospective. Gold standard: microbiology (n = 5); otherwise, overall clinical assessment, FU unknown.	Unknown.
	Kaim et al. [49]	2000	18/19*	3-phase ^99m^Tc-DPD bone scan, 740 MBq, γ-camera with 256 x 256 matrix.	No	Retrospective, highly selective patient group without orthopaedic implants or patients whose devices had been removed. Gold standard: microbiology (n = 13); otherwise, overall clinical assessment with minimum of 16 months FU.	Long-standing PTO, time interval between last surgical intervention and present study was 6.5 years (3 months – 39 years). Time interval between last surgery and imaging not clear.
	Meller et al. [52]	2002	19/21*	3-phase ^99m^Tc-MDP bone scan, 740 MBq. 150,000 – 400,000 counts for each projection.	No	Prospective. Gold standard: MRI (n = 19); histology/microbiology (n = 12); FU 1–6 months.	Symptoms of infection lasting for more than 6 weeks.
WBC (or AGA) scintigraphy	Ballani et al. [45]	2007	10	WBC scintigraphy with ^99m^Tc-HMPAO labelled autologous WBCs, 740 MBq, γ-camera with a 256 x 256 matrix (pixel size ∼ 2 mm). No late (24 h) phase scan.	No	Retrospective. Gold standard: microbiology (n = 5); otherwise, overall clinical assessment.	Unknown.
	Glaudemans et al. [47]	2013	49	WBC scintigraphy with ^99m^Tc-HMPAO labelled autologous WBCs, 500 MBq. Late phase scan included.	Yes	Retrospective. Gold standard: microbiology (n = 13), otherwise, overall clinical assessment at 6 months follow-up.	Unknown.
	Horger et al. [21]	2003	27/29*	AGA scintigraphy with ^99m^Tc labelled murine monoclonal antibodies. 750 MBq. Late phase scan included. γ-camera with 128 x 128 matrix.	Yes	Prospective. Gold standard: microbiology (n = 18); otherwise, overall clinical assessment with minimum of 6 months FU. 25 patients with suspected PTO (of which 1 non peripheral) and 2 (suspected) PJI.	Reactivation of chronic PTO suspected because of clinical inflammatory symptoms or elevated laboratory markers. Timeframe not specified.
	Kaim et al. [49]	2000	18/19*	AGA scintigraphy with ^99m^Tc labelled murine IgG antibodies, 555 MBq, γ-camera with a matrix of 256 x 256. Only one imaging time point (17 h).	No	Retrospective, highly selective patient group without orthopaedic implants or patients whose devices had been removed. Gold standard: microbiology (n = 13); otherwise, overall clinical assessment with minimum of 16 months FU.	Longstanding PTO, time interval between last surgical intervention and present study was 6.5 years (3 months – 39 years). Time interval between last surgery and imaging not clear.
	Meller et al. [52]	2002	19/21*	WBC scintigraphy autologous labelled with ^111^In, 18–37 MBq. Late phase scan included. 128 x 128 matrix, 250,000 – 500,000 counts for each projection.	No	Prospective. Gold standard: MRI (n = 19); histology/microbiology (n = 12); FU 1–6 months.	Symptoms of infection lasting for more than 6 weeks.
FDG-PET	Goebel et al. [46]	2007	50	^18^F-FDG-PET, 200 MBq. 128 x 128 matrix.	No	Prospective. Gold standard: microbiology (n = 50). 2 suspected trauma-related PJI included.	Symptoms of infection lasting for more than 6 weeks.
	Hartmann et al. [48]	2006	23	^18^F-FDG-PET, 300–400 MBq.	Yes	Retrospective, 15 patients with osteosynthesis, 3 prosthesis, 5 no material in situ. All trauma-related. Gold standard: microbiology (n = 23).	Symptoms of infection lasting for more than 6 weeks or presence of recurrent osteomyelitis.
	Meller et al. [52]	2002	19/21*	^18–^F-FDG-PET, 296 MBq.	No	Prospective. Gold standard: MRI (n = 19); histology/microbiology (n = 12); FU 1–6 months.	Symptoms of infection lasting for more than 6 weeks.
	Schiesser et al. [53]	2003	17/20*	^18^F-FDG-PET, 300–400 MBq.	No	Prospective. Gold standard: microbiology (n = 20), clinical FU 6 months.	Pain at motion or rest for at least 6 weeks, interval between last surgical intervention and FDG-PET scan 6 weeks – 14 months.
	Shemesh et al. [54]	2015	10	^18^F -FDG-PET, 296–555 MBq.	Yes	Retrospective. Gold standard: microbiology (n = 9, five cultures minimum); otherwise, overall clinical assessment with minimum of 1 year FU.	Time from initial surgery to PET/CT 2 months – 20 years.
	Wenter et al. [55]	2016	84	^18^F-FDG-PET, weight adapted dose (mean 252 +/- 76 MBq). 131 patients underwent PET/CT with mostly a full dose CT (n = 130) with iv contrast (n = 106).	No	Retrospective. Gold standard: microbiology (n = 143); otherwise, overall clinical assessment with minimum of 1 year FU. 215 patients, 192 suspected PTO (of which 12 non peripheral), 11 (suspected) PJI.	Causative event prior to PET scan dated back 12 ± 13 year in the clinically infected group and 10 ± 12 years in the clinically uninfected group.
			131		Yes		
MRI	Goebel et al. [46]	2007	18	No details on MRI technique provided	n/a	Prospective. Gold standard: microbiology (n = 18).	Symptoms of infection lasting for more than 6 weeks.
	Kaim et al. [49]	2000	18/19*	1.5-T MRI, slice thickness 3–10 mm. Both body coil (n = 10) and extremity coil (n = 8) used. All scans with iv gadolinium contrast.	n/a	Retrospective, highly selective patient group without orthopaedic implants or patients whose devices had been removed. Gold standard: microbiology (n = 13); otherwise, overall clinical assessment with minimum of 16 months FU.	Longstanding PTO, time interval between last surgical intervention and present study was 6,5 years (3 months – 39 years). Time interval between last surgery and imaging not clear.
CT scan	Goebel et al. [46]	2007	22	No details on CT technique provided.	n/a	Prospective. Gold standard: microbiology (n = 22).	Symptoms of infection lasting for more than 6 weeks.

* Presented as: number of patients/number of suspected peripheral PTO sites. Calculations are based on number of sites.

Abbreviations:

MDP: Methylene Diphosphate, DPD: 3,3-diphosphono-1,2-propanedicarboxyl acid tetrasodium salt, HMPAO: Hexamethylpropylene amine oxime, FU: follow up, AGA: anti-granulocyte antibodies, SPECT: Single-photon emission computed tomography, CT: Computerized tomography, T: Tesla, EANM: European Association of Nuclear Medicine

**Table 5:**
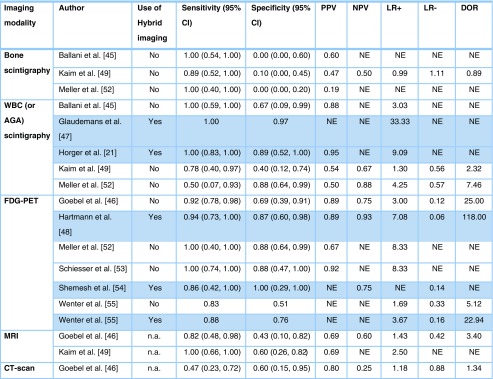
Diagnostic accuracy measures

Abbreviations: PPV: positive predictive value, NPV: negative predictive value, LR+: positive likelihood ratio, LR-: negative likelihood ratio, DOR: diagnostic odds ratio, NE: not estimable, n.a.: not applicable. The studies utilizing SPECT/CT or PET/CT are marked in blue.

### Three-phase bone scintigraphy

All three studies addressing the value of three-phase bone scintigraphy for diagnosing PTO are comparative studies [45, 49, 52]. Ballani et al. [45] compared three-phase ^99m^Tc-methylene diphosphate (MDP) bone scintigraphy with ^99m^Tc- hexamethylpropylene amine oxime (HMPAO) WBC scintigraphy. They studied a total of 24 patients of whom 10 patients were suspected of suffering from PTO; all TPBS results in this study were abnormal of which four were false positive. Kaim et al. [49] compared the value of combined TPBS/AGA scintigraphy with MRI for diagnosing PTO in a retrospective series with a highly selective patient group (19 suspected sites in 18 patients all with long-standing PTO). Meller et al. [52] performed TPBS as a selection tool for continuing with a WBC scintigraphy (which was subsequently performed in 28 patients of whom 19 had 21 suspected sites of PTO). All 19 PTO patients had a positive result, of which only 4 were true positive.

Overall, the sensitivity of TPBS was high (ranging from 89 to 100%), but the specificity was low (0 to 10%; Table 5). The other accuracy measures showed that bone scintigraphy without additional imaging has low diagnostic value for detecting PTO.

### WBC scintigraphy/AGA scintigraphy

The WBC scintigraphy and AGA scintigraphy studies are discussed together as both visualize the leukocyte infiltration within the patient. In WBC scintigraphy, the autologous WBCs of patients are collected, labelled ex vivo and subsequently reinjected. In AGA scintigraphy commercially available labelled monoclonal antibodies against the granulocytes are directly injected and bind in the patient to the leucocytes. Five suitable studies [21, 45, 47, 49, 52] were identified addressing the value of WBC or AGA scintigraphy (two studies combined with SPECT/CT [21, 47]) for diagnosing PTO. Ballani et al. [45] compared ^99m^Tc-HMPAO WBC scintigraphy with TPBS in a group of 24 patients with a clinical suspicion of osteomyelitis (of whom 10 were suspected with PTO). A limitation of this study is that their acquisition protocol consisted of a rather high dose of injected ^99m^Tc compared to current standards [47, 58] and they did not perform dual-time point imaging (images 2–4 h and 20–24 h after reinjection). Glaudemans et al. [47] described the results of ^99m^Tc-HMPAO WBC scintigraphy in a large retrospective study with 297 patients with various musculoskeletal infections (of whom 49 patients had suspected PTO). Labelling protocols were in accordance with current EANM guidelines [58] and scans were acquired correctly with imaging at two time points. Diagnosis was confirmed by microbiology in 13 cases. Clinical follow-up of at least 6 months confirmed mainly negative cases in all other patients (additional information obtained from the author).

A prospective study by Horger et al. [21] of 27 patients undergoing scintigraphy with technetium-99 m-labelled AGA combined with SPECT/CT in 25 patients for 27 suspected PTO sites (including one non-peripheral location) and 2 suspected PJI is reported. This focused specifically on the added value of CT with SPECT. Sensitivity was identical for WBC scintigraphy with SPECT alone and combined SPECT/CT (both 100%), whereas adding CT to SPECT improved the specificity from 78% to 89%. Kaim et al. [49], in a previously mentioned retrospective study, compared the validity of combined TPBS/^99m^Tc-labelled AGA scan with MRI for diagnosing PTO (18 patients, 19 infected peripheral sites). In this paper, the accuracy of the nuclear imaging was presented as a combined value for the TPBS and the AGA scan together. Again, imaging was only performed at one imaging time point (17 h after injection), which is a major limitation of this study. Finally, Meller et al. [52] reported on a comparative prospective study (^111^In WBC scintigraphy versus FDG-PET) with 30 consecutive chronic osteomyelitis patients of whom 19 PTO patients had 21 suspected infected sites in the peripheral skeleton.

Overall, sensitivity of WBC and AGA scintigraphy ranged from 50 to 100%, and specificity ranged from 40 to 97% (Table 5). LR+ ranged from 1.30 to 33.33 and LR- values of 0.56 and 0.57 were found. These results indicate strong to convincing diagnostic evidence of WBC and AGA scintigraphy to accurately detect, and weak evidence to exclude, PTO. However, one should bear in mind that the labelling procedures, acquisition protocols and interpretation criteria of the WBC/AGA scintigraphy might be different between some ‘dedicated’ centres, which can have some impact on the results. DOR values of 2.32 and 7.46 were calculated, showing that the odds of obtaining a positive test result was 2.32 to 7.46 times higher in a person with PTO than in a person without PTO. Additionally, the studies that used SPECT/CT in combination with WBC (or AGA) scintigraphy reported higher diagnostic accuracy.

### FDG-PET (/CT)

Six studies [46, 48, 52–55] were included addressing the value of FDG-PET in diagnosing PTO, three combined with CT [48, 54, 55]. Goebel et al. [46] prospectively investigated the diagnostic value of FDG-PET in 48 patients with peripheral PTO and compared this with CT (n = 22) and MRI (n = 18). Hartmann et al. [48] prospectively investigated 33 patients with FDG-PET/CT for suspected PTO, of which 23 had suspected PTO of the peripheral skeleton. Three patients in this study had a (suspected) trauma-related PJI. Meller et al. [52] prospectively compared FDG-PET with ^111^In WBC in 30 consecutive patients (of whom 19 were suspected of having peripheral PTO in 21 limbs) by using a dual-head coincidence camera. Schiesser et al. [53] prospectively analysed 17 patients with 20 suspected peripheral PTO sites using FDG-PET. Shemesh et al. [54] retrospectively looked at implant-related infections of the tibia in 10 patients investigated with FDG-PET/CT. Wenter et al. [55] reported the largest and most recent series of patients with PTO. They retrospectively reviewed the contributions of FDG-PET (n = 84) and FDG-PET/CT (n = 131) in a total of 215 patients with suspected PTO. If combined with CT, this was performed in the majority of patients with a full dose CT (n = 130) and with IV contrast (n = 106). The inclusion period was between 2000 and 2013; none of the patients had obvious signs of infection, 12 patients had suspected PJI and 12 non-peripheral suspected PTO sites were included.

Overall, sensitivity ranged from 83 to 100%, and specificity ranged from 51 to 100% (Table 5). The other measures showed moderate to strong diagnostic evidence of FDG-PET for either detecting or excluding PTO. Moreover, when the FDG-PET was combined with PET/CT, the diagnostic accuracy measures increased significantly.

### MRI

Two studies were included addressing the value of MRI in diagnosing PTO [46, 49], both with a comparative design. In the study of Goebel et al. [46], MRI [Tesla (T) strength not reported] was performed in 18 of 50 patients with suspected PTO. Kaim et al. [49] carried out a retrospective study comparing the value of a combined TPBS/AGA scan with a 1.5-T MRI for diagnosing PTO in a highly selective patient group (19 suspected sites in 18 patients all with long-standing PTO). All patients had T1-weighted images, 6/18 had T2-weighted images with fat suppression and 12/18 had T2-weighted images without fat suppression. All 18 had gadolinium enhancement. The third included study that describes the results of MRI for imaging PTO is the study by Meller et al. [52]. Unfortunately this study could not be included in this review for the results of the MRI because only seven patients with PTO of the peripheral skeleton underwent an MRI. Also, the authors used MRI as an adjudicator when no histology was available; therefore, sensitivity and specificity of the MRI for PTO was not evaluated in this paper and could not be calculated from the data given.

Overall, sensitivity values of 82 and 100% and specificity values of 43% and 60% were found in the studies of Goebel et al. [46] and Kaim et al. [49], respectively (Table 5). The other measures showed weak evidence of MRI for diagnosing or excluding PTO.

### CT

Only one study addressed the value of CT scanning in diagnosing PTO (Goebel et al. [46] ). Unfortunately, the technical aspects (number of slices and slice thickness) of the CT scan used in this study are not reported. For the 22 patients with suspected PTO who were analysed with CT, they found a sensitivity of 47% and a specificity of 60% (Table 5). The other measures showed weak diagnostic evidence of CT for diagnosing or excluding PTO.

## Discussion

Based on the best available evidence over the last 16 years, as presented in this paper, both WBC (or AGA) scintigraphy and FDG-PET have the best diagnostic accuracy for diagnosing or excluding peripheral PTO. The sensitivity for WBC (or AGA) scintigraphy ranged from 50 to 100%, and specificity ranged from 40 to 97%. For FDG-PET, this was 83 to 100% and 51% to 100%, respectively. Moreover, the studies, which combined the WBC/AGA scintigraphy with SPECT/CT [21, 47] or the FDG-PET with PET-CT [48, 54, 55] (which is in line with current practice) showed an increase in the diagnostic accuracy measures. For FDG-PET/CT, sensitivity ranged between 86 and 94% and specificity between 76 and 100%. For WBC scintigraphy + SPECT/CT this is 100% and 89 – 97% respectively. These results do partly concur with the previous reported accuracy on diagnostic imaging of chronic osteomyelitis by Termaat et al. [17]. They included in their meta-analysis papers published between 1975 and 2003 and favoured FDG-PET as the optimal imaging modality. However, studies included for FDG-PET consisted mainly of patients suspected of chronic osteomyelitis and not specifically PTO. Furthermore, in that era, almost no SPECT/CT or PET/CT camera systems existed and acquisition protocols especially for WBC scintigraphy have significantly improved since then [47, 58]. Glaudemans et al. [47] presented the results of a more recent large retrospective study including 297 patients with suspected bone or soft tissue infection of whom 49 PTO patients were analysed by WBC scintigraphy. Fourteen of the 49 PTO patients had a positive scan result and were, therefore, further analysed with SPECT/CT. For PTO, they found a sensitivity of 100%, a specificity of 97.4% and a diagnostic accuracy of 98%. Important to mention is that in this study, labelling protocols were in accordance with current EANM guidelines [58] and scans were acquired correctly with imaging at two time points which make these results more in accordance with current practice.

Choosing the most appropriate imaging technique for PTO remains difficult because there are advantages, disadvantages, pitfalls and contraindications of each option within the field of both nuclear medicine and clinical radiology. First of all, PTO is a condition that occurs in a very heterogeneous patient population. Limited mobility of the patient might not allow dual time point imaging and location of the infection, and co-morbidities and metal implants may affect the accuracy of the imaging techniques used. Secondly, what the surgeon needs to establish for proper pre-operative planning is not only the presence of an infection, but also whether there are specific features such as sequestra, cloacae, sinus tracts and intracortical or soft tissue abscesses present. This is also important in cases with no doubt about the diagnosis (for example, in patients with fistula or exposed metalwork) where imaging methods can be used with lower specificity and sensitivity for detecting PTO (such as an MRI scan). Thirdly, for pre-operative planning, it is important to determine fracture position, fracture union and to assess the integrity of implants. This is usually done by more conventional imaging methods which can sometimes be incorporated in the diagnostic workup of PTO (for example: a CT scan to assess fracture union can be omitted when a WBC scintigraphy with SPECT/CT is performed). All these factors need to be taken into account when ordering or advising a specific imaging technique. Establishing the diagnosis of infection is the first requirement for investigating PTO but, as mentioned before, imaging must also give information which allows planning of effective surgical treatment by defining the anatomical distribution of the infected or dead bone. The specific advantages and disadvantages of each imaging modality are summarized below.

Bone scintigraphy alone is not suitable for diagnosing PTO because of its low specificity, but it is relatively cheap and easy to perform with a high sensitivity. Therefore, in chronic cases with low suspicion of PTO, a normal bone scan can be used to exclude an infection.

WBC (or AGA) scintigraphy is a useful technique to diagnose PTO because leucocytes actively migrate to the site of infection and are, therefore, a more specific indicator for osteomyelitis. Also, the addition of the SPECT-CT allows better anatomical localisation and distinction between bone and soft tissue infections. A disadvantage is that performing a WBC scintigraphy is expensive, laborious and time-consuming (with strict labelling protocols and at least two scans on the two following days [19, 47, 58]).

FDG-PET is a relatively quicker whole-body imaging procedure (one imaging time point 60 minutes after injection) that can be used to detect multiple foci throughout the body. Disadvantages are that recent fractures and the presence of metallic hardware may decrease the accuracy of FDG-PET since FDG uptake will also be increased in inflammatory reactions [59]. Better spatial resolution and metal artefact reduction techniques have improved the quality of both MRI and CT over the last decade [60, 61] and the low costs, quick scanning time and availability make these scans an attractive first choice for many surgeons.

Plain X-rays and CT are specifically useful to image the degree of fracture union and to search for small sequestra, but are less suitable for determining the exact localisation of infected bone.

MRI can demonstrate the extent of bone and soft tissue involvement in cases of PTO but an absolute requirement is that both the surgeon and imaging specialist need to be experienced with interpreting the images in order to not be distracted by physiological changes (such as bone oedema) or accompanying normal tissue healing. The increasing use of internal fixation of fractures makes MRI less useful in the early diagnosis of PTO.

**Fig. 2:**
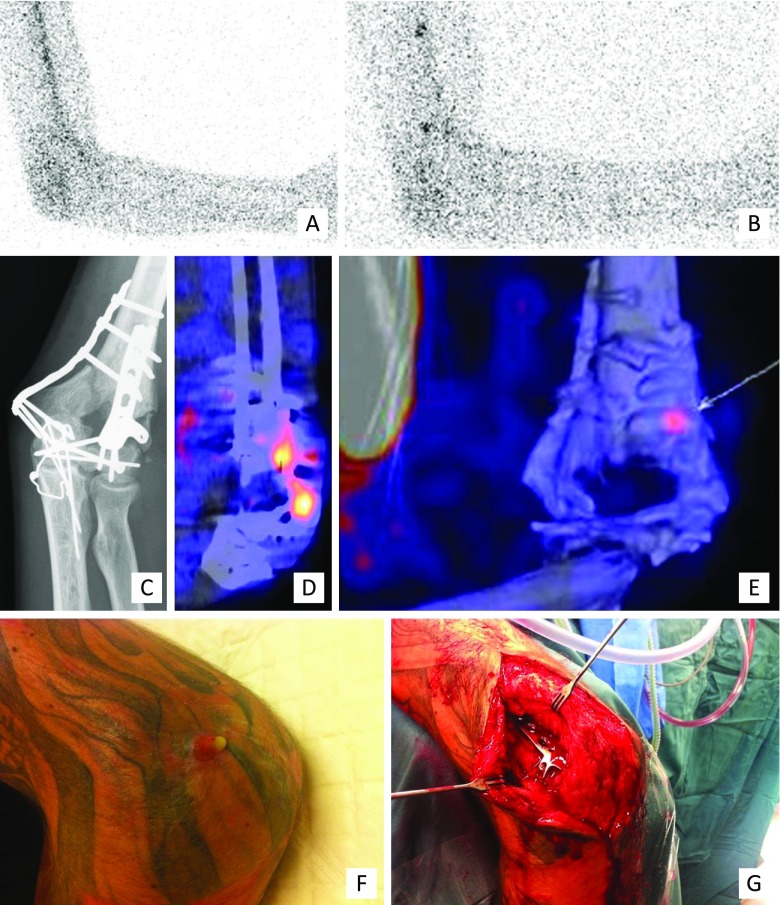
Clinical example of WBC scintigraphy + SPECT/CT. A 37-year-old man with a grade 3A complicated distal humeral fracture of the left elbow, initially treated with an external fixator and subsequently by plate osteosynthesis of the distal humerus. **c** X-ray: situation after recent fixation of the fracture with plate osteosynthesis, no signs of loosening or infection. After 4 months, he presented with a fistula and a clinical suspicion of osteomyelitis of the distal humerus. **a–b, d–e**: WBC scintigraphy (**a** image at 4 hours, **b** image at 24 hours, **d–e** fusion SPECT/CT images) after the injection of 220 MBq 99 m-Tc-labeled leucocytes demonstrated an infection around the implant at the lateral side of the elbow/distal screw. The low uptake points to only a low-grade appearance and the location to soft tissue involvement; this was confirmed at operation (**f** clinical pre-operative picture, **g** perioperative clinical picture)

**Fig. 3:**
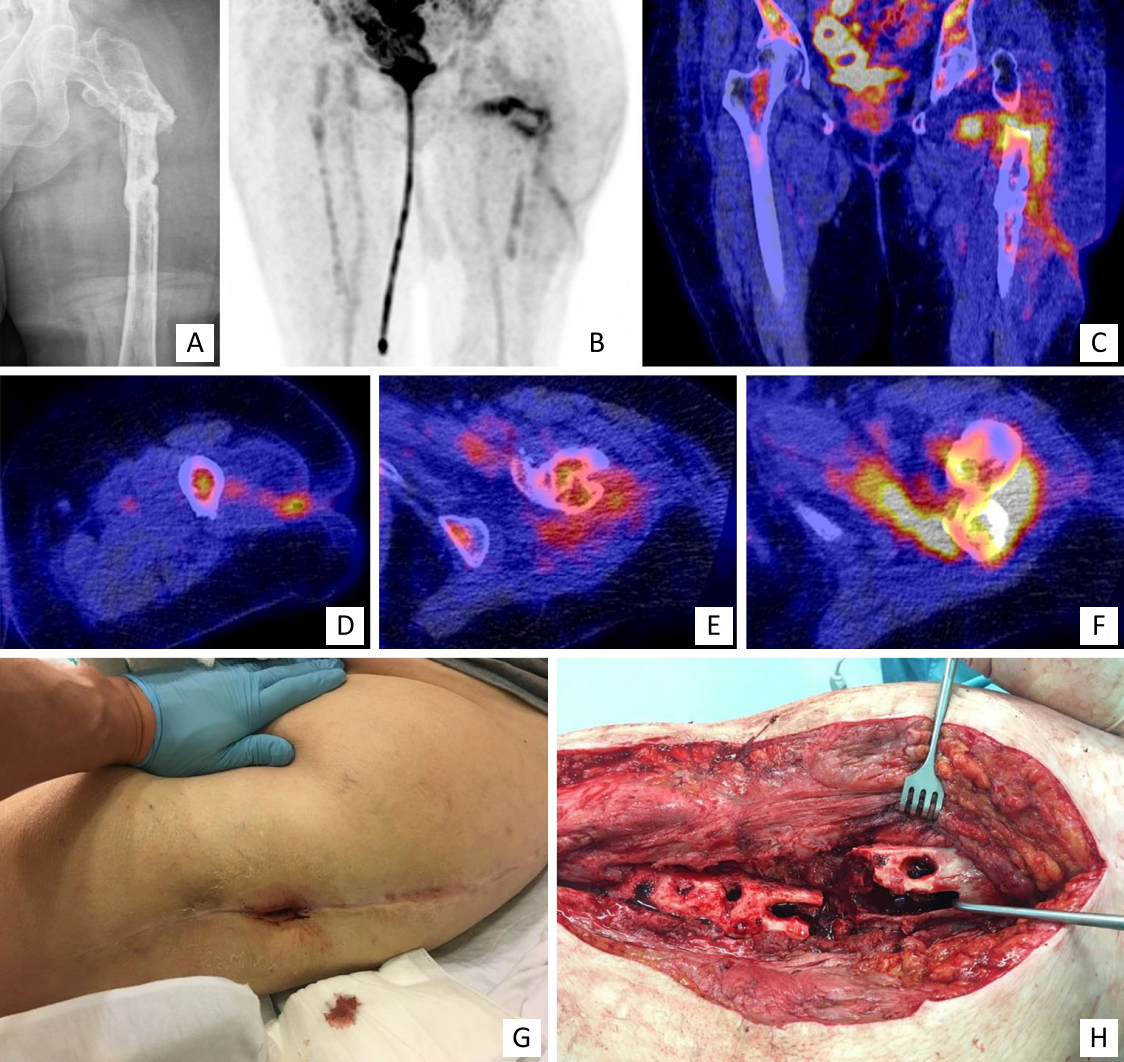
Clinical example of FDG-PET/CT. A 77-year-old woman who had a proximal femur fracture for which she underwent open reduction and internal fixation with a femur plate which had to be removed at a later stage due to infection. **a** X-ray, AP view: no consolidation, severe angulation, heterogeneous sclerotic aspect around the fracture. She was referred to our hospital with a fistula in the lateral thigh and a clinical suspicion of osteomyelitis of the proximal femur. Further imaging demonstrated an infection of the proximal femur, a medial abscess and a fistula coursing to the lateral aspect of the thigh which correlated with the clinical findings during surgery. **b–f** ^18^F FDG-PET/CT (**b** coronal FDG-PET image, **c** coronal fused FDG-PET/CT image, **d–f** transaxial fused FDG-PET/CT images). **g** clinical pre-operative picture, **h** perioperative clinical picture

**Fig. 4:**
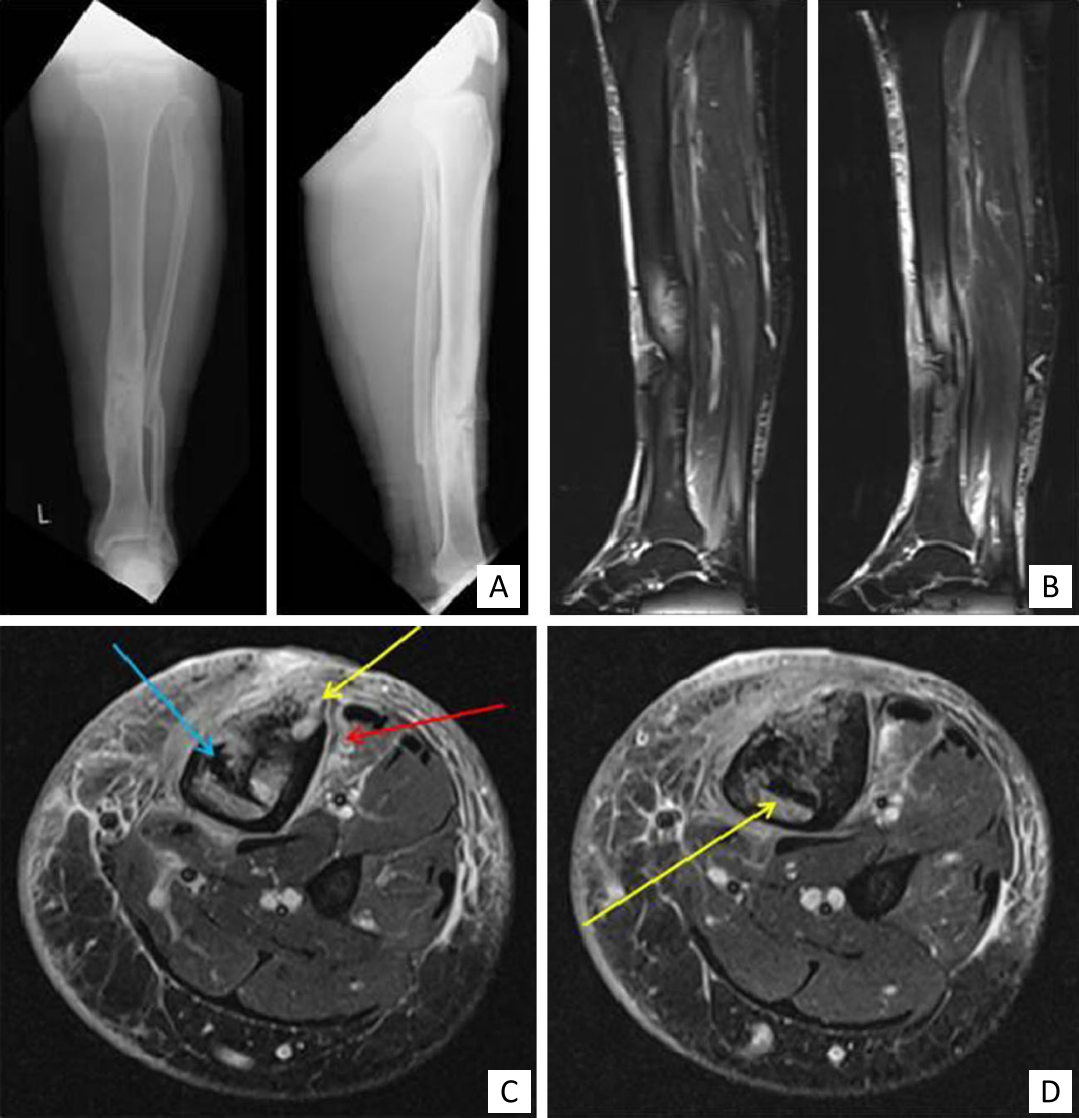
Clinical example of MRI. A 54-year-old man with a history of an open fracture treated with a plate many years ago. The fracture healed slowly and then the plate was removed because of continued skin breakdown over the front of the tibia. **a** Frontal and lateral radiograph demonstrating sclerosis and chronic periosteal reaction around the previous fracture site. **b** Sagittal fat-suppressed images of the calf demonstrating bone and soft tissue oedema. **c & d** Axial fat-suppressed images demonstrating sequestra (blue arrow), cortical abscesses (yellow arrows) and periostitis and soft tissue oedema (red arrow)

Clinical examples of the use of WBC scintigraphy + SPECT/CT, FDG-PET/CT and MRI for the surgical workup of patients with PTO are presented in Figs. 2, 3 and 4, respectively.

Clinicians need to be aware of the advantages and limitations of each imaging modality and the potential diagnostic accuracy. The issues of patient comfort, safety and personal experience of the surgeon and imaging specialist are of importance in choosing appropriate imaging techniques [12, 19, 59]. This review highlights the fact that the evidence in the literature is still limited and hampered by heterogeneous patient populations and quickly evolving imaging techniques. It is, therefore, clear that there is a need for further prospective studies on diagnostic imaging of PTO.

## Limitations of this study

Firstly, this study provides level 3 evidence on diagnostic imaging of PTO. The number of studies that could be included is limited, imaging techniques are heterogeneous and only four prospective studies met the inclusion criteria. Secondly, the studies were aimed at diagnosing or excluding PTO and did not focus on determining the anatomic distribution of infection for surgical planning. Thirdly, the studies provided limited information on the combination of hybrid imaging techniques such as SPECT/CT and PET/CT for detecting PTO and its extent.

## Conclusion

Based on the best available evidence of the last 16 years, both WBC (or AGA) scintigraphy combined with SPECT/CT or FDG-PET combined with CT have the best diagnostic accuracy for diagnosing peripheral PTO.
